# Acute effect of Ethanol and Taurine on frontal cortex absolute beta power before and after exercise

**DOI:** 10.1371/journal.pone.0194264

**Published:** 2018-03-14

**Authors:** Dailson Paulucio, Augusto Terra, Caleb G. Santos, Mauricio Cagy, Bruna Velasques, Pedro Ribeiro, Bruno M. da Costa, Mariana Gongora, Renato Alvarenga, Luciano Alonso, Fernando A. M. S. Pompeu

**Affiliations:** 1 Biometrics Laboratory, School of Physical Education and Sports, Federal University of Rio de Janeiro, Rio de Janeiro, Brazil; 2 Postgraduate in Physical Education, School of Physical Education and Sports, Federal University of Rio de Janeiro, Rio de Janeiro, Brazil; 3 Department of physiology in soccer, Botafogo de Futebol e Regatas, Rio de Janeiro, Brazil; 4 Army Biology Institute, Brazilian Army, Rua Francisco Manuel, Triagem, Rio de Janeiro, RJ, Brazil; 5 Biomedical Engineering Program, COPPE, Federal University of Rio de Janeiro, Rio de Janeiro, Brazil; 6 Brain Mapping and Sensory Motor Integration Laboratory, Institute of Psychiatry, Federal University of Rio de Janeiro, Rio de Janeiro, Brazil; 7 Neuroscience Laboratory of Exercise, Institute of Psychiatry, Federal University of Rio de Janeiro, Rio de Janeiro, Brazil; Hospital General Dr. Manuel Gea Gonzalez, MEXICO

## Abstract

Ethanol (ET) is a substance that modulates the Central Nervous System (CNS). Frequently, ET intake occurs combined with energy drinks, which contain taurine (TA), an important amino acid found in the body (i.e brain and muscles). Although TA administration has been used in the improvement of physical performance, the impact of TA, ET and exercise remains unknown. This study aimed to analyze the acute effect of 6g of Taurine (TA), 0.6 mL∙kg^-1^ of Ethanol (ET), and Taurine combined with Ethanol (TA+ET) ingestion on the electrocortical activity before and after a moderate intensity exercise in 9 subjects, 5 women (counterbalanced experimental design). In each of the 4 treatments (Placebo—PL, TA, ET and TA+ET), electroencephalography (EEG) tests were conducted in order to analyze changes in absolute beta power (ABP) in the frontal lobe in 3 moments: baseline (before ingestion), peak (before exercise) and post-exercise. In the PL treatment, the frontal areas showed decrease in ABP after exercise. However, in the ET+TA treatment, ABP values were greater after exercise, except for Fp1. The ET treatment had no effect on the Superior Frontal Gyrus area (F3, Fz and F4) and ABP decreased after exercise in Fp1 and Fp2. In the TA treatment, ABP increased after exercise, while it decreased at the peak moment in most of the frontal regions, except for Fp1, F3 and Fz. We concluded that after a moderate intensity exercise, a decrease in cortical activity occurs in placebo treatment. Moreover, we found a inhibitory effect of TA on cortical activity before exercise and a increased in cortical activity after exercise. A small ET dose is not enough to alter ABP in all regions of the frontal cortex and, in combination with TA, it showed an increase in the frontal cortex activity at the post-exercise moment.

## Introduction

Ethanol (ET), or Ethyl Alcohol, is a substance widely consumed by population; however, its effects on the Central Nervous System (CNS) are not completely known yet. ET is able to alter the CNS excitatory and inhibitory neurotransmitter balance, most times favoring the inhibitory influence [1]. The acute effect of ET contributes to a decrease in cortical activity [2]. Given the importance of such substance, its influence in some regions of the cortex (i.e. the frontal cortex) has been widely studied. This area takes part in functions such as learning, planning, decision making, motivation, attention, working memory and sensory and motor information integration [3]. The frontal region is meant to monitor performance and to implement necessary adjustments in cognitive control [4]. According to reports from the literature, ethanol may cause changes in some functions, such as for example executive control, decision making and risk management [5]. In addition to changes in the CNS, ET acute effect may also harm other organism functions, such as the metabolic and cardiovascular ones, and it may therefore negatively affect physical performance [2].

Ethanol intake commonly occurs together with energy drinks, which contain an amino acid called taurine (TA), which objective is to reduce the symptons of depression [6]. Due to the different components contained in energy drinks, it is difficult to affirm the effects of each substance interacting with ethanol. While caffeine is considered to be an excitatory substance, TA may act as a CNS inhibitor, just like alcohol, through the stimulation of inhibitory receptor agonists [7]. TA has been used in the treatment of alcoholism and to improve physical performance. In animals, the quantity of extra-cellular TA in the CNS was found to increase immediately after alcohol ingestion, thus suggesting a protecting effect of cerebral activity deriving from alcohol intake [8]. Whereas the CNS has multiple and complex functions, the combination of these two related substances and the exercise could clarify physiological mechanisms in response to neural stimulus. Although TA has been used as an alternative therapy [9] and as a performance enhancer during exercise [10], no studies have been conducted with human beings about the acute effects of such amino acid on electrocortical activity in the frontal cortex, when ingested alone or together with alcohol, before and after exercise.

Quantitative electroencephalography (qEEG) is a non-invasive tool of electrocortical activity analysis, and it has been widely used to assess the influence of physical exercise and of different substances in the central nervous system. After exercise, cortical activity may increase or decrease, depending on the physical exercise protocol and on the analyzed cortical region [11]. Beta frequency band has been used to evaluate the post-exercise effects, mainly in the brain frontal regions [11–13]. Nevertheless, the impact of the TA, ET and exercise on EEG activity remains unknown.

Our objective was to apply the qEEG in order to investigate the acute effect of ET, TA and both of these substances together in the frontal cortex before and after exercise. In particular, beta absolute power (13–30 Hz) was analyzed; this is a fast frequency and it is associated with the alertness/attention state and with motor activity. We hypothesize that the cortical activity (ABP) may be affected by these substances in the frontal cortex areas before and after exercise. Furthermore, TA may modulate ET effect when ingested together.

## Materials and methods

### Subjects

All participants, five healthy women (22 ± 3 years) and four healthy men (26 ± 5 years), were right-handed, regular drinkers of alcoholic beverages, amateur practitioners of physical exercise and non-smokers. The participants were asked to avoid physical activities with more than 5 metabolic equivalents (METs) and any food containing caffeine, alcohol and taurine for 48 hours before the test. Individuals with liver disorders(as determined by the activity of the enzymes aspartate amino-transferase, alanine aminotransferase, direct and indirect bilirubin, alkaline phosphatase, gamma-glutamyl transferase and lactatedehydrogenase) or who were taking medications, as well as alcohol users with a weekly intake greater than fifteen or less than two alcohol servings were excluded from this study. Volunteers read and signed an informed consent form. All experimental procedures were approved by the local ethics committee of University Hospital Clementino Fraga Filho (HUCFF) at Federal University of Rio de Janeiro (03899312.5.0000.5257). All of research protocols described here were carried out in accordance to the declaration of Helsinki".

### Experimental design

This study lasted approximately five weeks for each participant. The experiment began with an anthropometric and an effort test. During the following visits (once a week), a particular intervention was applied in randomized order (PL, TA, ET or TA+ET). The experimental design of these tests was in agreement with a specific treatment, using a protocol described previously [14]. Six grams of microcrystalline cellulose were diluted in 150 ml of an artificial orange juice (Clight^®^, 21 g.L^-1^) and used as the placebo (PL) solution, while six grams of powdered taurine (TA) were used for the experimental solution. Ethanol (Orloff^®^ vodka, Resende, RJ, Brazil—38% alcohol content) was administered in doses of 0.6 ml. kg^-1^, combined with the ingestion of artificial orange juice in a proportion of 2:1 (juice: vodka).

The TA and PL solutions were ingested two hours before the exercise, while the ET alcoholic beverage was ingested thirty minutes before the exercise. Such ingestion time was determined according to the peak plasma levels of each substance after intake [15]. In the TA+ET treatment, ethanol was ingested one hour and thirty minutes after the administration of taurine; Taurine administration followed a simple double-blind procedure. The EEG was recorded at the peak and post-exercise. Since the frontal region is related to executive functions, our aim was to observe the frontal regions dynamics in order to evaluate cognitive alterations when subjects were under drug influence.

### Ergometric protocol

A continuous and maximal load ramp testing on a cycle ergometer with an electromagnetic brake (Imbrasport^®^, Porto Alegre, RS, Brazil) was used to determine maximal power output (W_max_), maximal aerobic power $\left(\dot{V}O_{2max}\right)$ and anaerobic threshold (Vista Mini-CPX^®^, Vacumed^®^, Ventura, CA, USA).

The test started with the subject seated on a cycle ergometer for six minutes, followed by a four-minute warm-up pedaling without any load. After the warm-up, the maximum test was performed according to the procedures described by Nogueira and Pompeu [16]. The effort perceived exertion was determined at the end of each minute.

A constant-load protocol (SWT), with an intensity 10% lower than the load of the anaerobic threshold set for the maximum effort test was adopted for 10 minutes at a rate of 60 revolutions per minute (RPM) as a physical tool for the ergometric test, causing a stress on the body, in order to help in the analysis of ABP under the influence of PL, TA, ET and TA+ET.

### Blood collection—Biochemical analysis

Serum and plasma were obtained from peripheral blood samples at three moments (at the beginning, thirty minutes after ethanol ingestion and immediately after exercise) for each treatment (ET and TA+ET). Serum levels of liver enzymes were determined using the VITROS^®^ Chemical System and plasma Ethanol levels by Siemens Dimension^®^ Series.

### Acquisition of electroencephalographic signal

The EEG signal capture was performed using the BrainNet-BNT device 36 (EMSA, Medical Instruments, Brazil). Twenty monopolar electrodes were arranged following the 10/20 International System Protocol. The impedance of the electrodes was maintained between 5 and 10 kΩ. Recorded data had a total range of less than 70 μV. The data signal was amplified with a gain of 22,000, analogically filtered between 0.01 Hz (high-pass) and 80 Hz (low-pass), and sampled at 200 Hz. The *Data Acquisition* software (Delphi 5,0^TM^, USA) from the Brain Mapping and Sensory Motor Integration Lab was employed with the digital notch filter (60 Hz).

### Data processing and analysis

Visual inspection and independent component analysis (ICA) were applied to remove possible sources of artifacts produced during data collection. The data were collected using a bi-auricular reference, and they were transformed using the average reference after ICA was applied and artifact elimination was concluded. Through ICA and visual inspection, all the ranges which clearly showed artifacts such as blinking and muscle-related movements were removed. A classic estimator was applied for the power spectral density (PSD) performed by MATLAB 5.3 (Matworks, Inc.).

### Statistical analysis

Statistical analysis was performed using the Statistical Package for the Social Sciences^®^ (SPSS^®^ Inc., Chicago, IL, USA), SigmaPlot^®^ (Systat^®^ Software Inc., Chicago IL, USA) and Microsoft Excel^®^ for Windows^®^ (Microsoft^®^, Redmond, WA, USA). In order to analyze the absolute power, two-way ANOVA was used (treatment vs. moment) in order to assess the interaction of absolute beta power (ABP) between treatment and moment for all frontal electrodes. When an interaction between factors was found, a one-way ANOVA (Bonferroni post-hoc) was applied to each treatment for all frontal electrodes.

A ANOVA one-way was conducted to compare ethanol concentration in the plasma between moments (baseline, peak and post-exercise) to each treatment (ET and TA+ET). A Student t-test was used to compare ethanol concentration in the plasma moments in different treatments (ET Baseline vs. TA+ET Baseline; ET peak vs. TA+ET peak; ET post-exercise vs. TA+ET post-exercise).

Descriptive statistics were used with the mean ± standard error (se) (p ≤ 0.05). The p-value was adjusted through the bonferroni correction for multiple comparisons. The effect size was estimated using the Cohen’s d method.

## Results

When investigating the ethanol concentration in the plasma in ET and TA+ET treatments, a significant increase was seen for both treatments, compared to the Baseline values (0 mmol^.^L^-1^). However, there was no significant difference between baseline, peak and post-exercise to each treatment. Also, there was no significant difference between treatments when comparing the specific moments (ET Baseline vs. TA+ET Baseline; ET peak vs. TA+ET peak; ET post-exercise vs. TA+ET post-exercise) (Table 1).

**Table 1:** Plasma ethanol concentrations.

Treatment	Plasma ethanol concentration (mmol^.^L^-1^)			
	30 min after the ingestion	*P* value	Post-exercise	*P-*value
TA+ET	13.7 ± 2.9**	0.001	10.9 ± 1.4**	0.001
ET	12.3 ± 4.3**	0.001	9.7 ± 2.0 **	0.001

Values are expressed as means and SDs. Baseline values equal to 0 mmol^.^L^-1^.

** Significant difference compared to the baseline for each treatment.

A two-way ANOVA (moment vs. treatment) demonstrated interaction between the two factors for all investigated electrodes [Fp1- F(11;13795) = 7.156, η2p = 0,003, p<0.001; Fp2—F(11;13795) = 17.794, η2p = 0,008, p<0.001; F7—F(11;13795) = 26.174, η2p = 0,011; F8—F(11,13795) = 29.548., η2p = 0,013, p<0.001; F3—F(11,13795) = 22.903, η2p = 0,010, p<0.001; Fz—F(11,13795) = 18.800, η2p = 0,008, p<0.001; F4—F(11,13795) = 26.289, η2p = 0,011, p<0.001]. With the aim to analyze in detail all interactions found between moment and treatment, a one-way ANOVA was conducted separately for each treatment (PL, TA, ET and TA+ET). Interaction analyses are reported below. The effect sizes for all variables were summarized in S1 Table.

### Anterior prefrontal cortex (Fp1 and Fp2)

**Fig 1:**
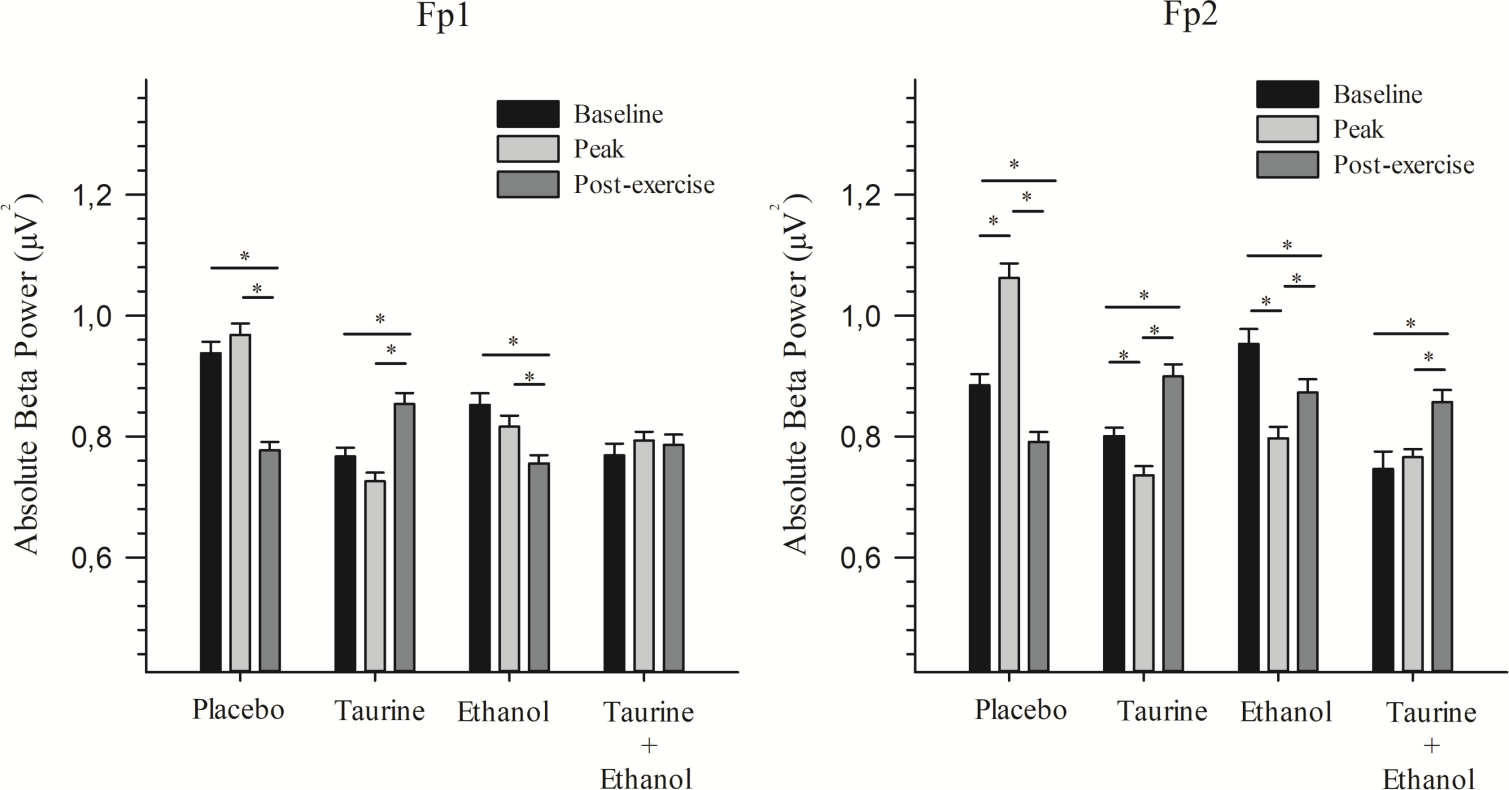
Absolute beta power of anterior prefrontal. Values are expressed as means and standard error. * Significant difference (p ≤ 0.05). This figure shows the absolute beta power of anterior prefrontal area (Fp1 e Fp2), in each treatment (Placebo, Taurine, Ethanol and Taurine + Ethanol) before (baseline and peak) and after (post-exercise) exercise.

Difference was found at Fp1 between the baseline and post-exercise moments (p<0.05) and between peak and post-exercise (p<0.05) for the PL, ET and TA treatments. On the other hand, at Fp2, difference was found among moments for all treatments. For the PL, ET and TA treatments, difference was observed among all moments (p<0.05). For ET+TA, significant difference occurred between the baseline and post-exercise (p<0.001) and between the peak and post-exercise (p = 0.009) moments (Fig 1).

### Inferior prefrontal gyrus (F7 and F8)

For the F7 electrode, difference was found between the baseline and peak (p<0.05), and between peak and post-exercise (p<0.05) moments for the ET and TA treatments. For the TA+ET treatment, difference occurred between the baseline and peak moments (p<0.001), and between baseline and post-exercise (p = 0.021). On the other hand, in the PL treatment, difference was observed between the baseline and post-exercise moments (p<0.001), and between peak and post-exercise (p<0.001).

**Fig 2:**
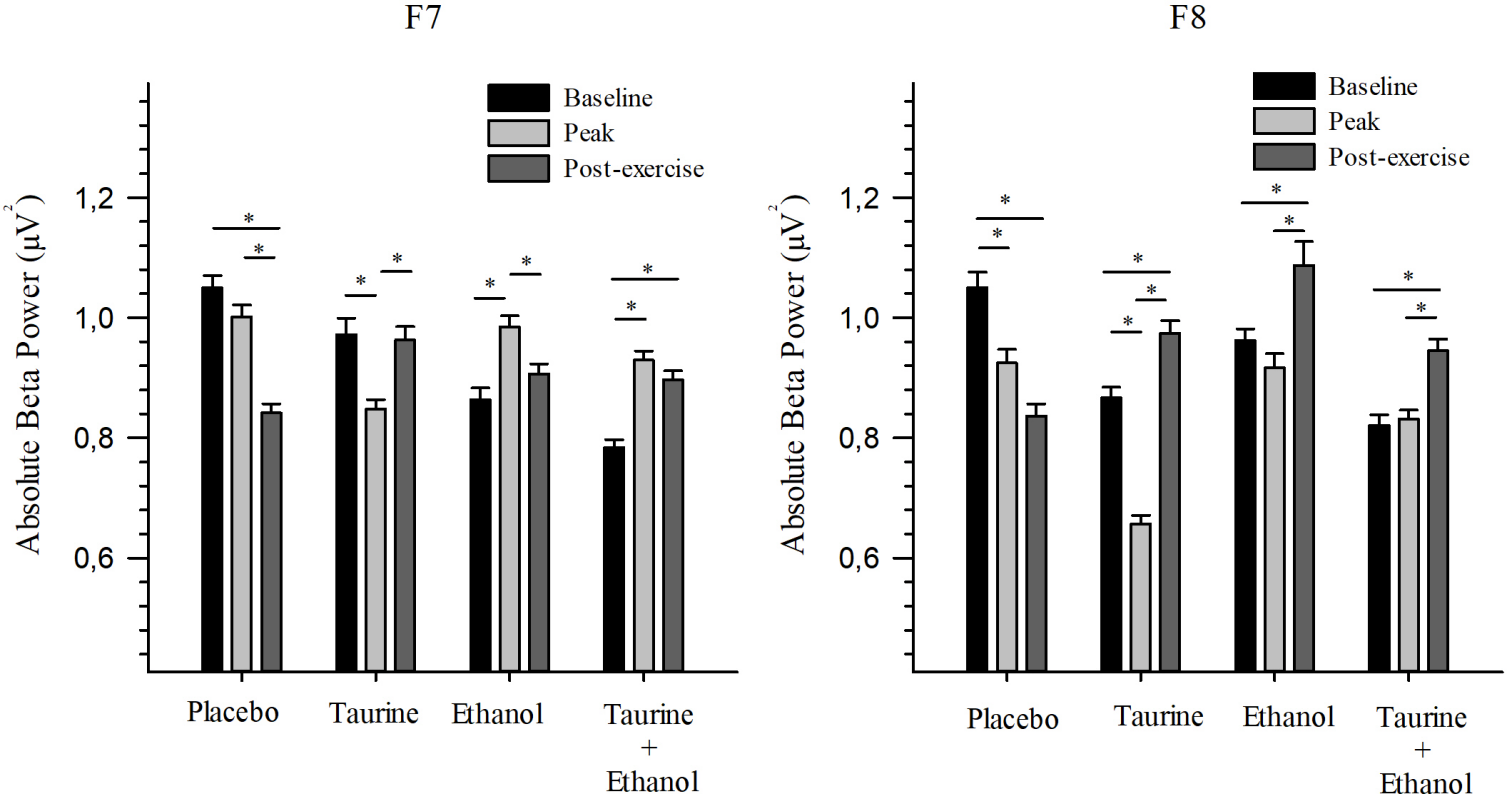
Absolute beta power of inferior prefrontal gyrus. Values are expressed as means and standard error. * Significant difference (p ≤ 0.05). This figure shows the absolute beta power of inferior prefrontal gyrus (F7 e F8), in each treatment (Placebo, Taurine, Ethanol and Taurine + Ethanol) before (baseline and peak) and after (post-exercise) exercise.

At F8, significant difference was found between the baseline and post-exercise moments (p<0.05) and between peak and post-exercise (p<0.05) for the ET and ET+TA treatments. For the PL treatment, significant difference was identified between the baseline and peak moments (p<0.001) and between baseline and post-exercise (p<0.001). And for the TA treatment, difference was found at all moments (p<0.05) (Fig 2).

### Superior frontal gyrus (F3, FZ and F4)

For the F3 electrode, significant difference was only found for PL and ET+TA. In the PL treatment, significant difference was observed between all moments (p<0.05); in the ET+TA treatment, difference occurred between the baseline and peak moments (p<0.001) and baseline and post-exercise (p<0.001).

For the F4 electrode, significant difference was found among all moments in the TA treatment (p<0.05). In the ET+TA treatment, significant difference was found between the baseline and post-exercise moments (p<0.001), and between baseline and peak (p<0.001). On the contrary, for the PL treatment, difference was observed between the baseline and post-exercise moments (p<0.001) and between peak and post-exercise (p<0.001).

**Fig 3:**
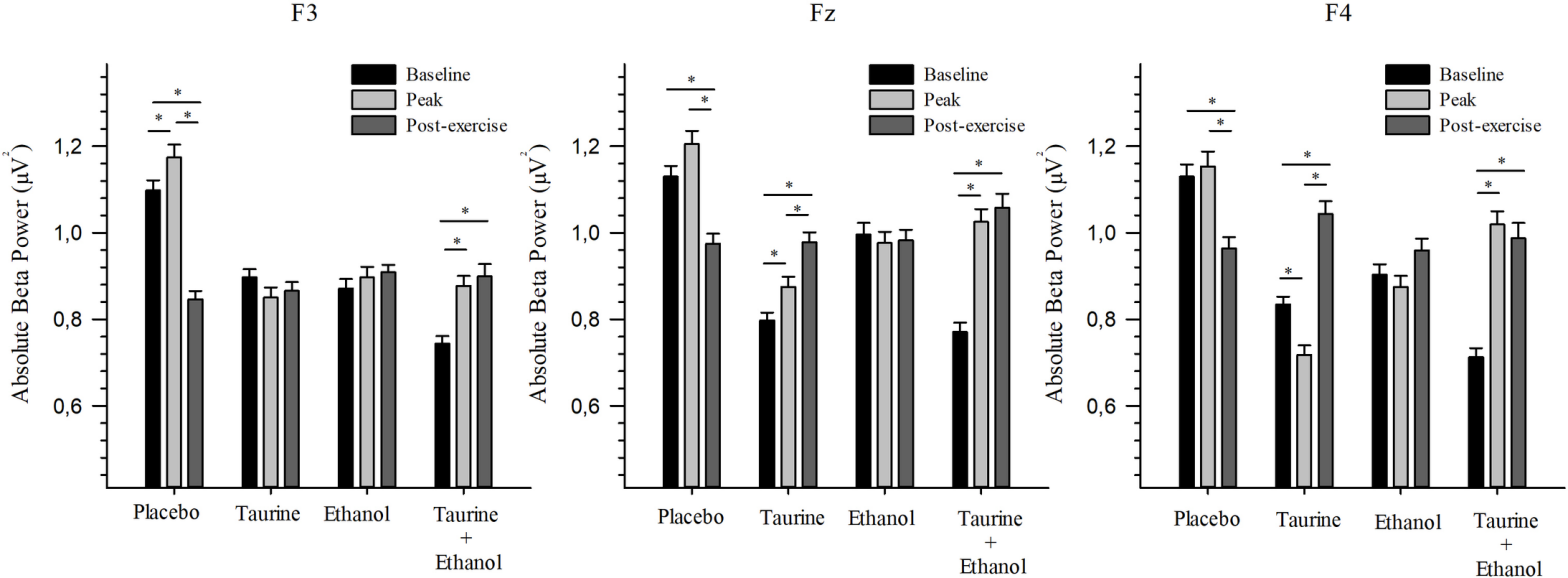
Absolute beta power of superior frontal gyrus. Values are expressed as means and standard error. * Significant difference (p ≤ 0.05). This figure shows the absolute beta power of superior prefrontal gyrus (F3, F4 e Fz), in each treatment (Placebo, Taurine, Ethanol and Taurine + Ethanol), before (baseline and peak) and after (post-exercise) exercise.

At Fz, just like at F4, significant difference occurred among all moments (p<0.05) for the TA treatment. Significant difference was found between the baseline and post-exercise moments (p<0.001) and between baseline and peak (p<0.001) for the ET+TA treatment. In the PL treatment, significant difference was observed between the baseline and post-exercise moments (p<0.001), and between peak and post-exercise (p<0.001) (Fig 3).

## Discussion

**Fig 4:**
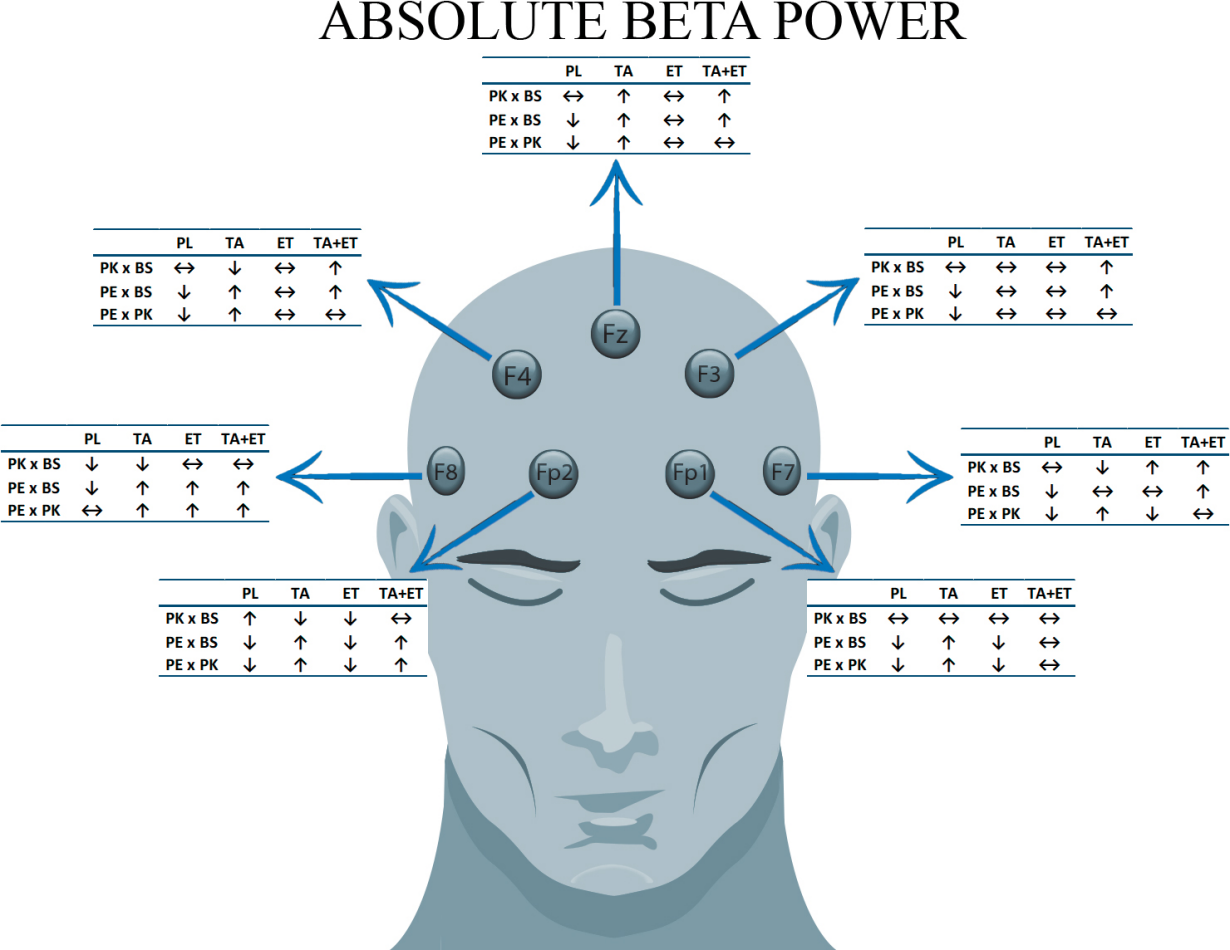
A cartoon representation demonstrates an overview of the absolute beta power results in different areas of frontal cortex. The arrow “**↑**” means an increase of absolute beta power. The arrow “**↓**” means a decrease of absolute beta power and the double arrow “**↔**” means no modification in absolute beta power.

The present study aimed to analyze the effect of TA, ET and these two substances administered together on the electrocortical activity, before and after moderate intensity exercise. An overwiew of these results was available as a cartoon (Fig 4). Specifically, changes in the absolute beta power in the frontal cortex were assessed. We hypothesize that the cortical activity (ABP) may be affected by these substances in the frontal cortex areas before and after exercise. Furthermore, TA may modulate ET effect when ingested together. Despite acknowledging the complexity of cerebral activity and of its multiple networks at various frequencies, due to methodological reasons, the discussion of electrophysiological data has been divided into three different subtopics, related to the areas associated with the investigated electrodes, as follows: Superior Frontal Gyrus—SFG (F3, Fz and F4); Anterior Pre-Frontal Cortex—PFC (Fp1 and Fp2); Inferior Frontal Gyrus–IFG (F7 and F8).

### Superior frontal gyrus—SFG (F3, Fz and F4) area—8 and 8l

Brodmann area 8 is considered to be an important area for motor learning. Studies from the literature have demonstrated this area to be responsible for starting, keeping, coordinating and planning complex movement sequences [17]. The transformation of such sensory signals into motor responses occurs through the visual and auditory systems [18]. In addition, this area also acts in working memory [19] and it may be involved in a cognitive control process, which is able to modulate the current emotional state [20].

As expected, no difference was found between the baseline and peak moments in the PL treatment for most of the electrodes relate to this region (i.e., Fz and F4), except in F3. However, a decrease in beta power was observed at the post-exercise moment, when compared to the others. Kubitz and Pothakos [21] have also observed a decrease in beta values after exercise. Nevertheless, Moraes et al. [12] and Bailey et al. [22] verified an increase in absolute beta power for F3 and F4 immediately after exercise. Kubitz and Mott [23] have not found any difference in absolute beta power before or after exercise. The different exercise intensity and duration and the time of cortical activity measurement may be involved with the different results. Such difference in the methods leads to different physiological responses (i.e. temperature, cerebral blood flow and hypoxia), which may influence the brain, therefore generating different results Lardon and Polich [24]. Bailey et al. [22] state that exercise intensity may affect cerebral activity: the greater the intensity, the greater the cerebral activity and, therefore, the beta values. The time of cortical activity measurement after exercise may also influence on beta values; in this same study, such values were found to be greater immediately after exercise, when compared to the pre-exercise moment, showing no difference between the pre- and post- moments when measured 10 minutes after exercise. Results by Petruzzello and Landers [25] also show a decrease in cortical activity along time for F4, when a comparison was done between the 10^th^ and 30^th^ minute after exercise.

In the TA treatment, difference was found for the Fz and F4 electrodes, with TA causing beta increase at Fz and beta decrease at F4. With regards to the post-exercise moment, an increase in beta power was found for both electrodes, when comparing this moment to the others. Taurine is used to improve exercise performance. Reports from the literature highlight some metabolic benefits for the membrane, fat oxidation, insulin sensitivity and cardiac muscle; however, its acute and chronic effects on the CNS need to be better investigated during exercise [10, 15] (Balshaw et al., 2013; Galloway et al., 2008).

Taurine ingestion may benefit the cardiac and skeletal muscles, by modulating the Calcium channels, therefore favoring cardiac contraction and helping with blood distribution [7]. Such greater cerebral blood flow, deriving from the response to TA ingestion, may influence greater cerebral activation (greater ABP values) at FZ and F4, after exercise [24].

It is important to highlight that no significant difference was found in the ET treatment for this region. Such findings are in agreement with the results in the studies by Ehlers et al. [26] and Cohen et al. [27], where no significant difference in ABP was found for the F3 and F4 electrodes (left and right side of this area, respectively), when comparing the moments before and after substance intake. Such responses may have occurred due to the similar ET quantity ingested in both studies mentioned in this research, with a consumption level between 0.6 and 0.8 ml/kg. In spite of such result, Zheng et al. [28] found ET to have an effect on the superior frontal gyrus, pointing out possible alterations in the CNS, such as: memory, motor control, cognitive ability and spatial function. The aforementioned authors observed changes in the alpha band values (lower frequencies) in this region, thus justifying the ET depressive effect in this area.

On the other hand, in the ET+TA treatment, similar results were found for the whole superior frontal gyrus region (F3, F4 and Fz), with an increase in ABP at the peak and exercise moments, when compared to the baseline moment. Since no changes in ABP were found in this region for the ET treatment, and since similar results were found for the TA treatment after exercise, TA is suggested to stand out, with similar physiological adaptations to the TA treatment during exercise, thus influencing beta values increase after exercise. No reports about the effect of both TA and TA+ET on this region are present in the literature.

Network changes in this region may cause impairment in motor and executive functions, working memory and high-demand cognitive functions [29]. Li et al. [30] observed a decrease in the grey matter volume in the SFG in patients with Parkinson’s disease, when compared to healthy individuals, suggesting a relation with the motor impairment typical of the disease. The greater ABP values found after exercise, in both TA and TA+ET treatments, when compared to baseline indicate greater cerebral activity after exercise in this region, with possible function improvement.

### Anterior pre-frontal cortex (Fp1 and Fp2)

The pre-frontal cortex (Brodmann area 10) has been associated with complex executive functions (executive control of behavior, decision making and inferential reasoning) and it plays an important role in memory control and manipulation, in addition to being also related to emotional and cognitive tasks [31]. On the other hand, beta is a fast frequency associated with attention processes, arousal and movement execution [32]. Our results highlighted an interaction between moment and treatment factors, that is, changes in the beta cortical activity in this region will occur according to the ingested substance and to the treatment moment (i.e., baseline, peak or post-exercise). Specifically, when investigating the left anterior pre-frontal cortex (Fp1) and the right pre-frontal cortex (Fp2), the TA and ET conditions were found to have an effect on the right pre-frontal cortex during the performance peak, with no difference between the baseline and peak moments in the left pre-frontal cortex for all conditions. In particular, all substances caused a decrease in beta power during its performance peak. As observed for ABP, changes in the right hemisphere may be related to the substance effects on some specific functions of this area. The anterior pre-frontal cortex is directly related to visuospatial prospective memory. In addition, the ABP oscillations are associated with motor activity, thus we hypothesized this frequency band to be involved in keeping the current sensory/cognitive state [32].

This region is strictly related to risk decision making; according to Kwon et al. [31], risk decisions are more related to the right side of this region, suggesting these decisions to be dependent on the level of activation of this side. On the other hand, Aron et al. [33] state the right side to be responsible for suppressing memories and for responding to visual and auditory distractions.

According to Arnsten [34], the PFC is crucial to centralize attention onto a specific task and to regulate behavior and emotions, especially to inhibit inappropriate emotions, impulses and habits. This region is also necessary for goal planning as well as behavior and thought organization. Mainly the right hemisphere is important to regulate distractions, inappropriate behavior and emotional response.

As with regards to specific treatments, decrease in ABP was found in the PL treatment for the post-exercise moment, when compared to the baseline moment. The different intensities and duration of exercise have an influence in the acute physiological adaptations, which could interfere in the electrocortical activity. For example, catecholamines and temperature regulation, also modulated by the hypothalamic nucleus, may influence beta activity [35]. However, more studies are needed to better understand the mechanisms inherent to the relation between different exercise protocols and beta activity in this specific region.

When analyzing the ET+TA combination, a significant effect was found only in the right anterior pre-frontal cortex, with ABP increase at the post- moment, when compared to the others. Our results suggest the intake of the ET+TA combination alone do not provoke any changes in the anterior pre-frontal cortex; however, exercise potentializes the combination of these two substances, thus causing significant beta power increase in the right anterior pre-frontal cortex. As previously said, this area is directly related to executive control, specifically motor and behavioral executive control, attention, and inhibitory control. In particular, the right hemisphere features characteristics related to spatiality and development of direction sense. Therefore, this study hypothesized beta power increase at Fp2 to be associated with an increase in attention and executive motor control, required for the performance of physical exercise.

Despite a beta decrease at the peak moment, TA provokes an increase in beta power during the exercise moment in both hemispheres of the anterior pre-frontal cortex, when ingested alone. Therefore, depending on the region, taurine may decrease electrocortical activity; this finding is in agreement with other studies demonstrating the inhibitory effect of this amino-acid on the central nervous system [36]. However, the acute effect of taurine seems to be related to the effect of exercise, with an increase in beta activity after exercise. Some studies found taurine to also increase glutamate receptor activity, through the regulation of cytoplasmic and intramitochondrial calcium regulation [37]. Even when taurine is ingested with alcohol, ABP increased after exercise for Fp2. Since beta plays an initial role in the selective inhibition of some actions and in cognitive control [38], it is possible to suggest that TA may have two important effects: first, it may diminish beta activity and negatively alter the two aforementioned tasks; secondly, it may increase cortical activity and, consequently, beta values, thus improving the performance of such tasks.

In the ET treatment, this substance was found to also play an important role in the right anterior pre-frontal cortex; however, in addition to the ET intake alone decreasing ABP, the combination of ET and exercise also provokes decrease in beta power. Such results are in agreement with the literature about ethanol effects before and after intake, with lower cortical activity caused by the acute effect of the substance [39] mainly at Fp2 [40]. Such electrocortical activity decrease in this region may negatively influence some processes, such as working memory and decision making [5]. This may happen due to the ethanol effects on the CNS, directly facilitating GABA neurotransmitter transmission and inhibiting the glutamate neurotransmitter functions [1].

Few studies have related beta band to this region (PFC). Researches in the literature performed with animals (monkeys) affirm that beta neuronal synchronization involves execution tasks, such as rule selection. A given rule is represented by a group of PFC neurons, oscillating in synchrony at this frequency [41]. Buschman et al. [42] also demonstrate beta band synchrony in the PFC of monkeys to select the most relevant group of rules.

Studies with humans highlight beta oscillations to start the cognitive control and selective inhibition process in the PFC [38]. In other words, specifically in this region, the alcohol effects are found and suggested to significantly influence inhibition decrease. Empirically, it is possible to observe people who consume alcohol in bars and restaurants becoming more extroverted and disinhibited and acting in a certain way, only because they were under the influence of such substance.

### Inferior frontal gyrus–IFG (F7 and F8)

Traditionally, the IFG has been widely associated with aspects related to language and semantics [43]. In addition to this, the activation of such area may be related to selection tasks and working memory (spatial and non-spatial) [19]. Activities in this area may also reflect the inhibition of a motor act associated with a reactive emotional response [20]. Increase in beta activity may be associated with increased cortical activity, and according to Gilbertson et al. [44], beta increase may be related to worse movement performance, demonstrating triggered voluntary movements to be smaller when beta activity is greater. However, the ability to perform a Go/NoGo task was associated with the increase in beta band activity in the IFG [45]. In this region, interaction between the moment and treatment factors was also found for the two analyzed electrodes.

In the PL treatment, decrease in ABP occurred at the baseline moment, compared to the post-exercise moment in this area. Schneider et al. [13] have also observed similar results after moderately intense running, that is, exercise causes decrease in absolute beta power. A study by Li et al. [11] is in agreement with such results; they identified a decrease in the left IFG cortical activity after exercise.

On the other hand, in the ET+TA treatment, increase in ABP was found for the peak moment, when compared to baseline only at F7, demonstrating this treatment to influence beta power only in the left IFG. However, beta increase was verified at the post-exercise moment for both left and right IFG, showing an influence of exercise on absolute beta power in both hemispheres. Despite acamprosate (a substance containing TA) being used in the treatment of alcoholics in order to reduce ethanol-abstinence symptoms Plosker [46], there are no studies in the literature about the acute effect of TA+ET on the electrocortical activity in humans. Taurine seems to maximize the effects of alcohol in the left hemisphere of the IGF, considering that beta activity stayed high after exercise in the TA+ET treatment.

On the other hand, in the right hemisphere, the TA effect together with ET did not modify beta activity, considering that the TA+ET results were similar to the ET ones. In great part of the frontal cortex, TA seems to increase beta activity after exercise, even with ET intake. We hypothesized the increase in cortical activity after exercise to be related to the TA effects on the modulation of the glutamate receptor activation, through the cytoplasmic and intramitochondrial calcium regulation [47]. In a study with animals, the acute administration of TA+ET, TA was proved to exert a neuroprotecting action on the apoptosis induced by the acute effect of ethanol on the cerebellum [48]. The pioneer present study allows verifying the cortical activity of the TA+ET acute administration in humans, but the mechanisms inherent to the effects of such substances on the CNS need to be furtherly studied.

In the ET treatment, a substance influence on the left hemisphere of this area (F7) was found, provoking an increase in beta power. However, when ET is combined with exercise, an increase in beta power is found in the right hemisphere (F8), while decrease in beta power is found in the left hemisphere (F7) of this region. The area correspondent to the right hemisphere (F8) is activated when cognitive tasks require inhibition [49]. In addition to the function related to language, the IFG activation may occur during the execution of distal movements, such as grabbing, and is also directly related to mirror neurons for expressive movements [50]. We suggest that some of these specific functions related to the IGF may have been influenced by alcohol intake and exercise, seen through changes in beta activity.

Therefore, it is possible to observe this small dose of ET to alter the left IFG activity, thus possibly affecting some specific functions of this region, such as attention [19]. In terms of TA treatment, TA was seen to provoke a decrease in beta power in both hemispheres. Furthermore, in TA treatment, absolute beta power increased in the right side of this region after exercise, thus showing interaction between such amino acid and exercise. Such effect may interfere in some tasks associated with this region, since the right inferior frontal gyrus is directly related to some specific brain functions, such as inhibitory and attention control [51]. The absolute beta power decrease at the peak moment may be explained by its already proven inhibitory effects [47].

Studies involving the effect of TA have been increasing in recent years due to their potential physiological influence. In addition, the simultaneous use of TA and ET justifies a better understanding of the acute effects of these substances. The complexity of CNS and the multiple, distinct, and important functions that each of its regions play, generate innumerable interpretations of brain functions. Thus, the combination of two substances that appear to be metabolically associated, and exercise as a physiological stressor, provide a more comprehensive understanding of the relationship of those compounds and the operation of CNS.

Although our work has shown attractive results, some limitations could be highlighted: the amount of ET administered in comparison to some studies was small. It would be applicable to evaluate these effects in higher ET concentration. The taurine peak levels were defined from literature data. The analysis of taurine serum levels could avoid possible problems involving individual variability of this measure and also improve the explanation of the results. It is a fact that counterbalanced design favors the number of cell cases for each condition and, consequently, the sample size. However, evaluating a larger number of subjects and the gender effect would be interesting due to the great interindividual variability of neural measures.

In summary, due to many different results, we can highlight that the acute effect of placebo ingestion decreased the electrocortical activity after exercise. The TA acute effect seems to inhibit the superior frontal region at the plasma peak; however, in such region, the TA effect increases ABP after exercise. A small ET dose was not enough to alter beta activity in the superior frontal area and it was sufficient to diminish it on the left side of the brain (Fp1 and F7) after exercise. We could observe that the ingestion of taurine in combination with ethanol showed different results of the ingestion of ethanol separately. TA when ingested together with ET had an similar effect in the right side (Fp2, F8, F4) of the frontal cortex that TA treatment after exercise. More research is thus needed to better understand the mechanisms involved in the CNS response, such as electrocortical activities and frontal cortex functions, to such substances (TA and ET) with different dosage, exercise type and intensity.

## Supporting information

S1 Table The effect sizes for variables of frontal cortex, moments and treatments. (DOCX)
