# Supplementary material for: Acute effect of Ethanol and Taurine on frontal cortex absolute beta power before and after exercise
Source: PLoS One. 2018 Mar 14;13(3):e0194264. doi: 10.1371/journal.pone.0194264 (PMC5851630; doi:10.1371/journal.pone.0194264)
Supplement: S1 Table — (DOCX) [file pone.0194264.s001.docx]

|  |  | Anterior Pre-Frontal Cortex (d) | | Inferior Prefrontal Gyrus (d) | | Superior Frontal Gyrus (d) | | |
| --- | --- | --- | --- | --- | --- | --- | --- | --- |
|  |  | FP1 | FP2 | F7 | F8 | F3 | Fz | F4 |
| **Placebo** | Baseline - Peak | 0.05 | 0.26 | 0.07 | 0.25 | 0.11 | 0.08 | 0.02 |
|  | Baseline - Post-exercise | 0.27 | 0.16 | 0.33 | 0.34 | 0.34 | 0.19 | 0.18 |
|  | Peak - Post-exercise | 0.34 | 0.42 | 0.27 | 0.13 | 0.42 | 0.26 | 0.18 |
| **Taurine** | Baseline - Peak | 0.08 | 0.13 | 0.17 | 0.39 | 0.07 | 0.11 | 0.17 |
|  | Baseline - Post-exercise | 0.15 | 0.17 | 0.01 | 0.16 | 0.05 | 0.25 | 0.25 |
|  | Peak - Post-exercise | 0.23 | 0.27 | 0.17 | 0.51 | 0.02 | 0.13 | 0.36 |
| **Ethanol** | Baseline - Peak | 0.06 | 0.21 | 0.19 | 0.06 | 0.03 | 0.02 | 0.03 |
|  | Baseline - Post-exercise | 0.17 | 0.10 | 0.07 | 0.11 | 0.06 | 0.02 | 0.06 |
|  | Peak - Post-exercise | 0.11 | 0.11 | 0.13 | 0.15 | 0.02 | 0.01 | 0.09 |
| **Taurine + Ethanol** | Baseline - Peak | 0.04 | 0.03 | 0.31 | 0.02 | 0.20 | 0.30 | 0.36 |
|  | Baseline - Post-exercise | 0.03 | 0.13 | 0.24 | 0.21 | 0.20 | 0.31 | 0.28 |
|  | Peak - Post-exercise | 0.01 | 0.16 | 0.07 | 0.20 | 0.03 | 0.03 | 0.03 |

S1 Table - The effect sizes for variables of Frontal Cortex. moments and treatments.
